# Decreased decorin expression in the tumor microenvironment

**DOI:** 10.1002/cam4.231

**Published:** 2014-03-17

**Authors:** Benedek Bozoky, Andrii Savchenko, Hayrettin Guven, Fredrik Ponten, George Klein, Laszlo Szekely

**Affiliations:** 1 Department of Microbiology, Tumor and Cell Biology (MTC), Karolinska InstitutetStockholm, Sweden; 2 Department of Immunology, Genetics and Pathology, RudbecklaboratorietUppsala, Sweden

**Keywords:** Decorin, matrix, proteoglycan, tumor microenvironment, tumor stroma

## Abstract

Decorin is a small leucine-rich proteoglycan, synthesized and deposited by fibroblasts in the stroma where it binds to collagen I. It sequesters several growth factors and antagonizes numerous members of the receptor tyrosine kinase family. In experimental murine systems, it acted as a potent tumor suppressor. Examining the Human Protein Atlas online database of immunostained tissue samples we have surveyed decorin expression in silico in several different tumor types, comparing them with corresponding normal tissues. We found that decorin is abundantly secreted and deposited in normal connective tissue but its expression is consistently decreased in the tumor microenvironment. We developed a software to quantitate the difference in expression. The presence of two closely related proteoglycans in the newly formed tumor stroma indicated that the decreased decorin expression was not caused by the delay in proteoglycan deposition in the newly formed connective tissue surrounding the tumor.

## Introduction

Decorin is a small stromal proteoglycan belonging to the small leucine-rich proteoglycan (SLRP) gene family. It is mainly synthesized and deposited by fibroblasts and was named after its tight binding to collagen I fibers that it decorates 1–3. It has a 396 amino acid long core domain (decoron) with several leucine-rich repeats that create a curved solenoid fold 4. One molecule of decoron has the capacity to interact with four to six collagen molecules 5 and thus plays an important role in the regulation of fibrillogenesis 6–9. It sequesters multiple growth factors, including transforming growth factor beta-1,2 (TGF-*β*1, -2), and myostatin 10–13 and directly antagonizes several members of the receptor tyrosine kinase (RTK) family including epidermal growth factor receptor (EGFR), insulin-like growth factor receptor I (IGF1R) and hepatocyte growth factor receptor (HGFR) 14–17. Decorin also participates in the control of inflammation through binding to the toll-like receptors 2 and 4 18. Mouse model experiments have shown that disruption of decorin expression can increase intestinal tumor formation 19 and accelerate lymphoma formation 20. In vitro findings have shown that decorin expression by fibroblasts is reduced when they are grown together with prostate cancer cell lines 21. Due to its tumor suppressive potency, decorin was called a “guardian from the matrix” in a recent review 22.

The tumor microenvironment, or tumor stroma, makes up the milieu in which the tumor exists. It is normally made up of cells commonly present in normal connective tissue, including fibroblasts, endothelial cells, pericytes, and immune cells. Decorin expression in different tumor stroma has not been extensively studied and reported findings are somewhat contradictory. According to some studies it was upregulated in pancreatic cancer compared to normal pancreatic tissue 23,24. Decorin's expression has also been shown upregulated around Kaposi sarcoma's 25. Another study showed that it was downregulated in lung cancer 26. Decorin expression was also studied at different stages of breast cancer development where it showed progression dependent decrease of the staining signal adjacent to the malignant cells 27. Examining the Human Protein Atlas online database we have surveyed decorin expression in silico, in several different tumor types. We have used the same approach previously to identify new cancer-associated fibroblast signatures 28. Considering the postulated role of decorin as a tumor suppressor, we expected to find it expressed in normal tissues but not in the tumor stroma. In view of the possibility that an immature/not fully formed connective tissue may lag behind in proteoglycan deposition, we have also examined the expression of three closely related SLRPs—asporin, biglycan, and osteoglycin. They were selected as the closest reported relatives of decorin included in the Human Protein Atlas online database.

## Material and Methods

The Human Protein Atlas 29,30 is an online database (http://www.proteinatlas.org) containing over 10 million images of immunostained human tissue samples. It provides data on the protein expression patterns of various cell types in both cancerous and normal tissues. The current version, 11.0, offers data for 15,156 genes with protein expression profiles based on 18,707 antibodies. Each antibody in the database has been used for protein profiling on tissue and cell microarrays containing normal human tissues (144 individuals), cancer tissues (216 patients), and 50 cell lines. Tissue microarray sections are counterstained with hematoxylin, enabling a nonspecific visualization of microscopic structures, in addition to the binding of the antibody that results in a dark-brown stain 31. All tissue samples were collected from anonymized surgical specimens, in accordance with approval from the local ethics committee. The included cancer types represent the 20 most common forms of human cancer, for example, breast cancer, prostate cancer, lung cancer, etc. Included samples from cancer specimens represent a typical mixture of the different subtypes of cancers with an effort to include high- and low-grade malignancies when appropriate. For each antibody a validation score is provided, indicating how well the quality assurance data support the specificity of the antibody toward the expected human target protein 30. The validation is based on immunohistochemistry, immunofluorescence, western blot, and protein array where applicable. The scores can be supportive, uncertain, or nonsupportive.

Using the Human Protein Atlas online database, we investigated decorin expression in different normal and cancerous tissues. Immunohistochemistry, western blot, and protein array validations were all supportive for the used antibody, HPA003315.

We have developed a software (Protein Expression Quantifier) to quantitate the expression. It extracts the DAB signal from the Human Protein Atlas images. After inversion of the image it uses an automatic threshold algorithm to define DAB-positive particles. These objects are counted and quantitated automatically. The extracted data were tabulated as a surface plot and in chart form. An additional image analysis was made using the Human Protein Atlas to investigate the expression patterns of asporin, biglycan, and osteoglycin in skin and breast tumors compared with normal tissues.

## Results

**Figure 1:**
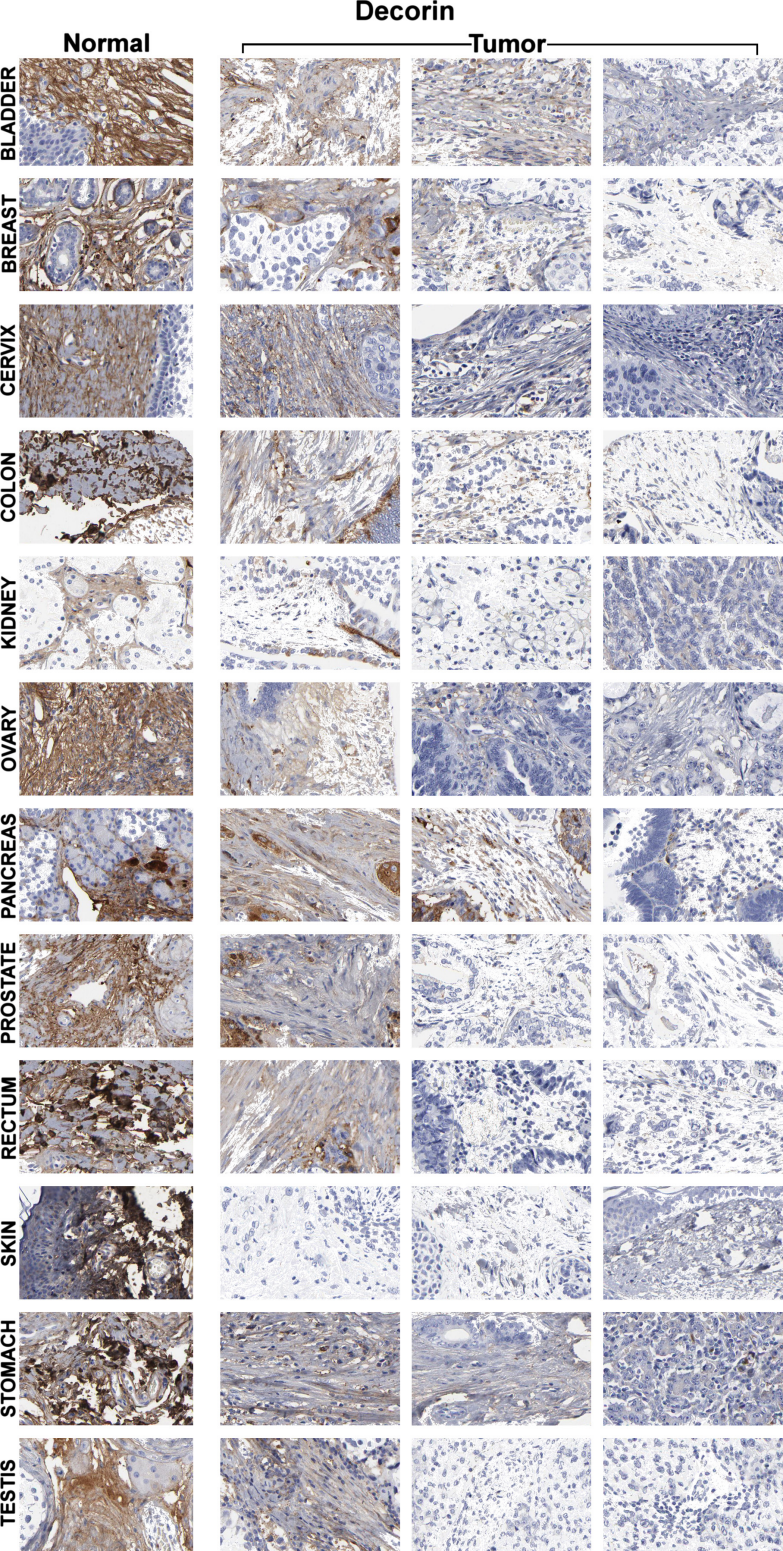
Decorin comparison between different tissues. Images were taken from the Human Protein Atlas (http://www.proteinatlas.org) online database. Each row represents a different tissue and each individual image in the montage represents a tissue sample from a different patient. The first column shows normal tissues whereas the last three columns show the corresponding tumor samples. Decorin is strongly expressed in the stroma of the normal tissues but its expression is significantly reduced and even absent in the tumor samples.

Decorin expression was compared in human urinary bladder, breast, cervix, colon, kidney, ovary, pancreas, prostate, rectum, skin, stomach, and testis tissues (Fig. 1). The following tumor types were analyzed, with sample sizes indicated in bold, surrounded by square brackets after each tumor type: urothelial carcinoma of the urinary bladder **[23]**, lobular **[7]** and ductal **[15]** carcinoma of the breast, squamous cell carcinoma **[17]** and adenocarcinoma **[6]** of the cervix, adenocarcinoma **[10]** of the colon, adenocarcinoma **[23]** of the kidney, serous **[13]** or mucinous **[3]** cystadenocarcinoma and endometrioid **[5]** carcinoma of the ovary, adenocarcinoma **[20]** of the pancreas, low **[7]**, medium **[4]**, and high **[7]** grade adenocarcinoma of the prostate, adenocarcinoma **[11]** of the rectum, squamous cell carcinoma **[11]** and basal cell carcinoma **[11]** of the skin, adenocarcinoma **[23]** of the stomach, and embryonal carcinoma **[10]** and seminoma **[8]** of the testis.

**Figure 2:**
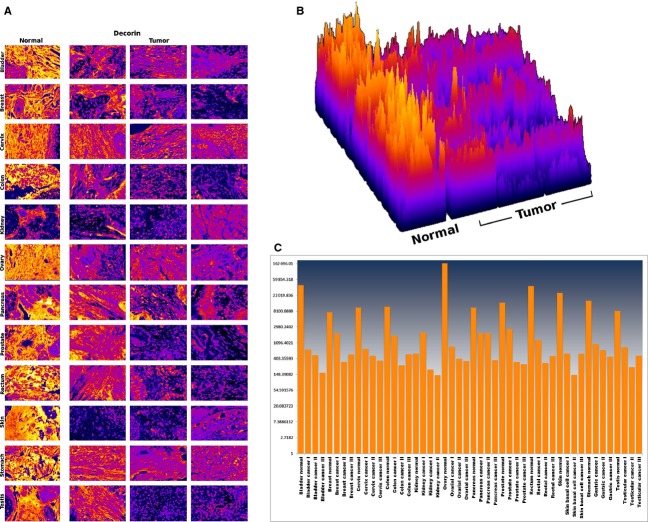
(A) Inverted DAB signal from Figure 1 quantified by a self-tailored software. (B) Surface plot of the inverted DAB signal from A. (C) Inverted DAB signaling presented in chart form. Each peak represents a normal tissue sample. Decorin in strongly expressed in the normal tissues less in tumor samples. Note differences between individual tumors derived from the same tissue.

We found that decorin was abundantly expressed in the stroma of all examined normal tissues and absent or significantly reduced in the corresponding tumors. Each individual image in the montage represents a different surgical specimen from a different patient. Images were taken from the Human Protein Atlas online database (http://www.proteinatlas.org). Different tumors derived from the same tissue differed with regard to the level of reduction (Fig. 1, last 3 columns). Quantitation of the image analysis is shown in Figure 2A–C. Figure 2A shows the inverted DAB signal of Figure 1. The measured signal was then represented in a surface plot (Fig. 2B) and as a chart (Fig. 2C). The peaks in Figure 2C represent the normal tissues, showing a strong decorin expression. Individual differences can also be distinguished between tumors of the same tissue.

**Figure 3:**
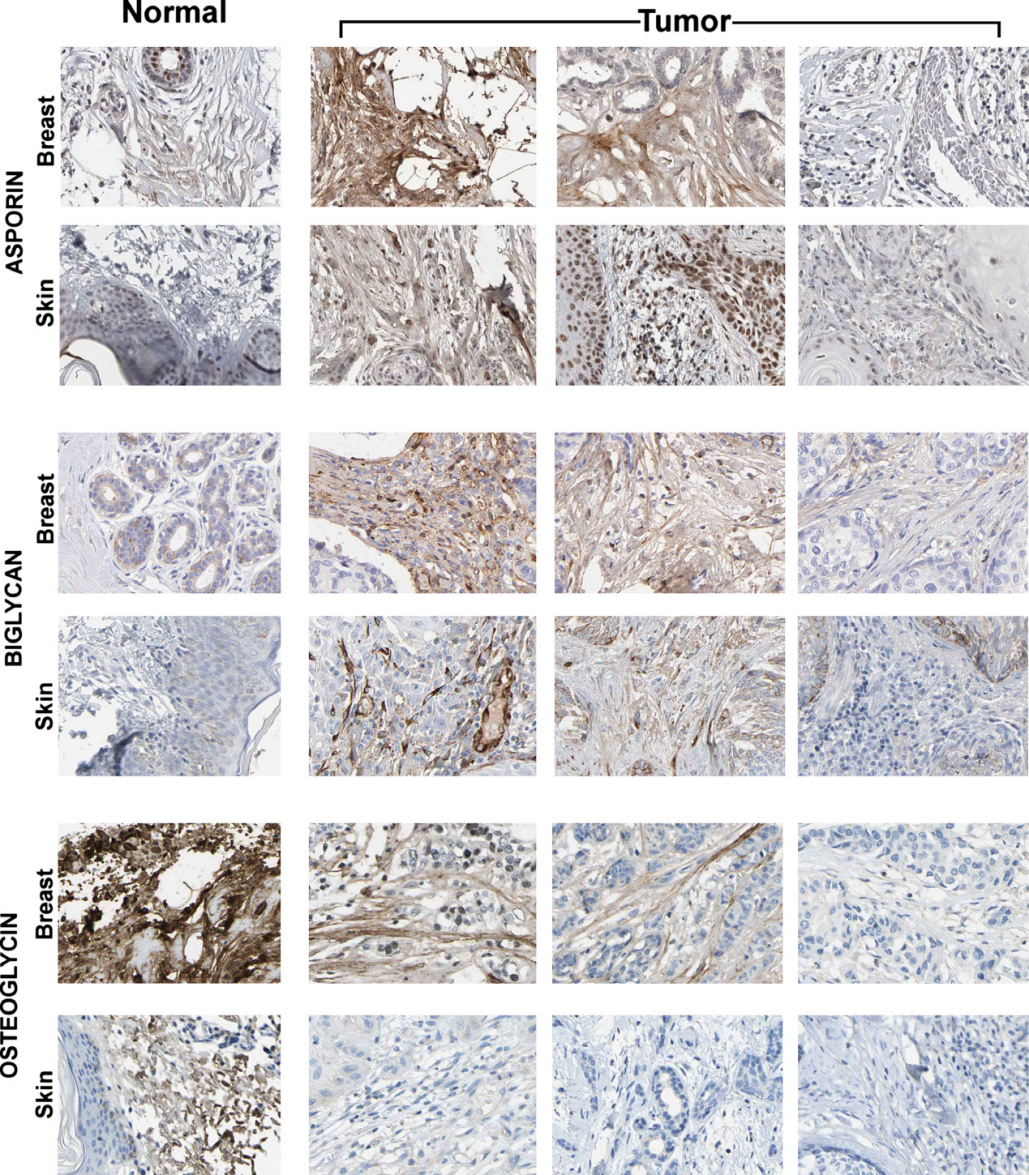
Montage showing the difference in expression of three additional small leucine-rich proteoglycans (SLRPs): asporin, biglycan, and osteoglycin in normal and tumor stromata. Each image in the montage represents a tissue sample from a different patient. The first column shows normal skin or breast tissues. The last three columns show the corresponding tumor samples. Like decorin, osteoglycin expression is high in normal stroma and decreased in the tumor samples. Asporin and biglycan show the opposite pattern, negative in normal tissues but often expressed in the tumor stromata.

The closely related proteins asporin, biglycan, and osteoglycin were analyzed in breast and skin tissues (Fig. 3). Like in Figure 1, each individual image in the montage represents a different surgical specimen, each from different individuals. The first column represents normal tissues and the last three columns represent the corresponding tumor tissues. Similarly to decorin, osteoglycin was expressed in normal connective tissue and decreased in the tumor stroma. In contrast asporin and biglycan was not expressed in normal tissues but was found to be expressed in the tumor stroma.

## Discussion

The Human Protein Atlas online database of immunostained tissue sections permits the study of protein expression in a wide range of normal and tumor samples. It also gives information on the antibody validation by immunohistochemistry, western blot, and protein array. Our self-developed software (Protein Expression Quantifier) provided an objective support of our findings.

Our analysis revealed that decorin is abundantly secreted and deposited in normal connective tissue, but its expression was consistently decreased in the tumor microenvironment. Previous studies have shown a decreased expression of decorin in the stroma of specific tumor types 26,27. Our results are in line with these results while they contradict some other ones 23–25. The inconsistency could be explained by the different methods to detect decorin expression, like the use of different antibodies, and by the analysis of different tumor tissues (e.g., Kaposi Sarcomas). Among the three closely related proteoglycans, osteoglycin showed a similar pattern whereas asporin and biglycan did not.

Decorin has been identified as a tumor suppressor in experimental murine systems 19,20. It was also shown that fibroblasts express lower levels of decorin when cultured in presence of cancer cells 21. Our findings of a substantially decreased expression in the tumor associated, compared with normal connective tissue is in line with this view. The presence of two closely related SLRPs in the tumor stroma indicated that the decreased decorin expression was not caused by the delay in proteoglycan deposition in the newly formed connective tissue surrounding the tumor. Our findings are in line with the possibility that a decreased decorin expression in the tumor microenvironment facilitates tumor growth and progression. This raises the question whether differential decorin expression in different tumor samples may have clinical, prognostic significance.

Changes in the connective tissue within and around tumors are increasingly recognized as important contributors to tumor development and progression. Decorin and the closely related deposited proteoglycans showed a clear, marked difference in stromal expression. This emphasizes their potential role in carcinogenesis. Suggested mechanisms for its tumor-suppressive ability include 1 directly binding and downregulating EGFR 32, 2 interfering with angiogenesis 33, and 3 inhibiting cell migration and growth by suppressing *β* catenin levels 17. An additional mechanism could be attributable to decorin's collagen-binding property. Naked collagen stimulates tumor growth in vitro 34–37. It could be therefore surmised that decorin could antagonize tumor growth by binding to collagen, covering the collagen surfaces that are responsible for the stimulatory effect. Downregulation of decorin in the tumor stroma may facilitate tumor invasion to neighboring tissues.
